# Development and Validation of an LC-MS/MS Assay to Quantitate 2′,4′,6′-Trihydroxyacetophenone in Rat and Dog Plasma and its Application to a Pharmacokinetic Study

**DOI:** 10.3390/molecules25194373

**Published:** 2020-09-23

**Authors:** Hee Jo Yoo, Se-Jung Hwang, Jeong-Hun Lee, Wang-Seob Shim, Yun-Woong Choi, Sang Min Cho, Eun Kyoung Chung, Jun-Bom Park, Kyung-Tae Lee

**Affiliations:** 1 Department of Pharmaceutical Biochemistry, Kyung Hee University, Seoul 02447, Korea; heejoyoo93@gmail.com (H.J.Y.); ztztzt08@hanmail.net (J.-H.L.); 2 Department of Life and Nanopharmaceutical Sciences, Graduate School, Kyung Hee University, Seoul 02447, Korea; 3 Department of Pharmacy, College of Pharmacy, Kyung Hee University, 26 Kyungheedae-ro, Dongdaemun-gu, Seoul 02447, Korea; teuerjung@naver.com; 4 Kyung Hee Drug Analysis Center, Kyung Hee University, 26 Kyungheedae-ro, Dongdaemun-Gu, Seoul 02447, Korea; wsshimm@naver.com; 5 Korea United Pharmaceutical Company, Seoul 06116, Korea; choi0528@kup.co.kr (Y.-W.C.); sweety1723@kup.co.kr (S.M.C.); 6 College of Pharmacy, Sahmyook University, Seoul 01795, Korea

**Keywords:** 2′,4′,6′-trihydroxyacetophenone, LC-MS/MS, validation, pharmacokinetic study, polo-like kinase 1

## Abstract

In the present study, a simple, rapid, and reliable bioanalytical method was developed using liquid chromatography with tandem-mass spectrometry (LC-MS/MS) to quantify 2′,4′,6′-trihydroxyacetophenone (THAP) in rat and dog plasma with 2′,4′,6′-trihydroxybenzaldehyde as an internal standard (IS). The LC-MS/MS instrument was operated in the multiple reaction monitoring (MRM) mode to detect THAP at m/z transition 166.89 > 82.8 and IS at 152.89 > 82.8, respectively. A simple, one-step protein precipitation (PP) method was employed with acetonitrile for sample preparation. Utilizing a Gemini C18 column, THAP and IS were separated with an isocratic mobile phase consisting of 10 mM ammonium acetate and methanol (10:90, *v/v*) at a flow rate of 0.2 mL/min. Total chromatographic run time was 2.5 min per sample injection. The standard calibration curve for THAP was linear (*r^2^ ≥* 0.9987) over the concentration range of 0.1 to 100 µg/mL with the lower limit of quantitation (LLOQ) of 0.1 µg/mL (S/N ratio > 10). According to the regulatory guidelines from the U.S. Food and Drug Administration (FDA) and the Korea Ministry of Food and Drug Safety (MFDS), our newly developed biomedical analytical method was fully and adequately validated in terms of selectivity, sensitivity, linearity, intra- and inter-day precision and accuracy, recovery, matrix effect, stability, and dilution integrity. Our validated assay was successfully utilized in a nonclinical pharmacokinetic study of THAP in rats and dogs.

## 1. Introduction

**Figure 1:**
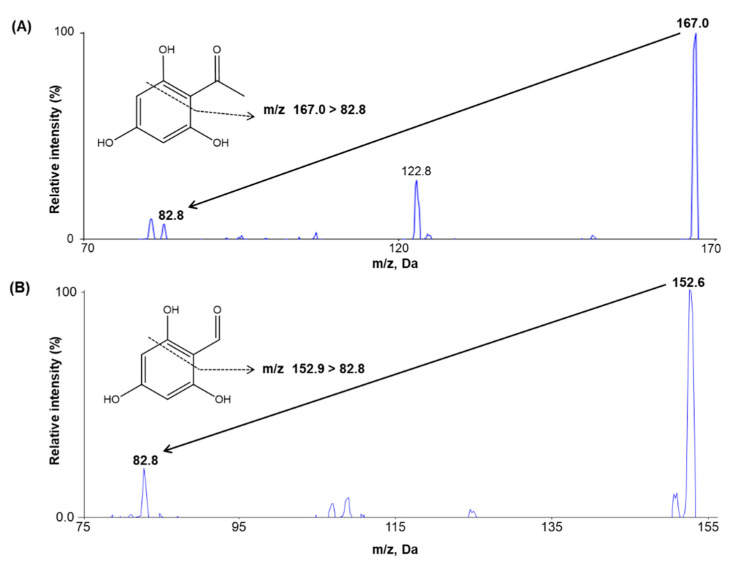
Product ion mass spectra and the fragmentation pattern of (**A**) 2′,4′,6′-trihydroxyacetophenone (THAP) and (**B**) trihydroxybenzaldehyde (internal standard, IS).

2′,4′,6′-Trihydroxyacetophenone (THAP) is a benzenetriol in which hydrogen atoms on the phenyl group are replaced with hydroxyl groups at the 2, 4, and 6 positions (Figure 1). According to previous studies, THAP has been suggested as an active ingredient of several dietary supplement products [1,2]. It is often isolated from *Myrcia multiflora* (Myrtaceae) and acts as a pancreatic lipase inhibitor to delay intestinal absorption of dietary fat, showing antiobesity and mixed lipid-lowering effects [3,4]. The cholesterol-lowering effect of THAP was also due to its potent choleretic activity through enhanced excretion of bile acid and cholesterol by inducing mRNA expression and activity of CYP7A1 in animals [5,6]. This choleretic activity is mediated by the effects of THAP and one of its metabolites (glucuronide conjugation) on the multidrug resistance protein-2 (Mrp2) transporter [7]. In addition, THAP has been detected as the aglycone part of acetophenone glycoside from *Curcuma comosa* Roxb. which is one of the widely consumed dietary supplement products in Europe and Southeast Asia [8]. Previous studies suggested THAP isolated from *C. comosa* to be a promising compound for the treatment of hormone-resistant breast cancer as a polo-like kinase 1 (Plk1) inhibitor [9,10]. Plk1 inhibitors including THAP may have targeted therapeutic activity to treat Plk1-overexpressed cancer [11,12]. These various pharmacologic activities make THAP a promising candidate compound to be developed as a drug with further investigation.

Previous studies suggested potential inhibitory effects of THAP derivatives on proliferating human tumor cell lines. The reported IC_50_ values were > 10 × 10^−5^ M, which is equivalent to 1.68 μg/mL of THAP [13]. The potential anticancer activity of THAP through Plk1 inhibition has increased the interest in evaluating its drug-like properties from absorption, distribution, metabolism, and excretion (ADME) to develop as a novel targeted drug. However, the minimum effective concentration and maximum tolerated dose of THAP have not been investigated yet in either nonclinical or clinical settings. According to the regulatory guidance [14], a broad range of concentrations of the potentially pharmacologically active compound should be evaluated to establish the concentration-response relationship. In this context, several different doses should be tested in animals of different species to produce variable in vivo concentration-time profiles. This information from nonclinical studies is critical to designing an initial safe dose as well as the clinically relevant testing dose range of a drug-like candidate compound such as THAP for future clinical studies. With the recent increase in the popularity of using THAP as a dietary supplement globally, its drug-like properties should be better characterized for potential development as a novel drug product.

In order to evaluate the drug-like properties, primarily ADME, an accurate and precise bioanalytical method is critical to elucidate the concentration-time profile of the compound in both nonclinical and clinical studies. To our knowledge, no quantitative analytical method has been developed for THAP and thus, the dose-concentration relationship of THAP has never been established in vivo. Therefore, the objective of our present study was to develop and fully validate a simple, accurate, reliable, and sensitive biomedical analytical assay using liquid chromatography coupled to tandem mass spectrometry (LC-MS/MS) to quantify THAP in rat and dog plasma. The developed analytical method was employed to measure THAP concentrations in rat and dog plasma after oral administration of THAP in a nonclinical pharmacokinetic study.

## 2. Results and Discussion

### 2.1. Method Development

The MS detection conditions for THAP and trihydroxybenzaldehyde (internal standard, IS) were optimized in the negative ion mode because of minimal interference and sufficient reproducibility in the analyte response compared to the positive ion mode. After individually infusing THAP and IS into the mass spectrometer with a syringe pump at a flow rate of 10 µL/min, THAP and IS were detected at the mass transition ranges of 166.89 > 82.8 and 152.89 > 82.8, respectively, as shown in the product ion mass spectra for both THAP and IS (Figure 1). Next, the optimal acquisition conditions for product ions were determined to improve signal intensity by manually varying the ionization source settings. The optimized source temperature and ion spray voltage were 500 °C and −4.500 V, respectively, resulting in the highest signal intensity among the tested conditions. Similar chromatographic behavior was seen for both the analyte and IS without endogenous interference.

Utilizing the optimized MS conditions, several chromatographic conditions such as the column, column temperature, flow rate, and mobile phase composition were evaluated for minimal matrix effects caused by endogenous substances as well as optimal peak shape, chromatographic separation, and run time. In the present study, the following different columns were investigated to optimize chromatographic separation of THAP and IS: 1) Phenomenex Luna^®^ C18 column (50 × 2.0 mm, 3 μm), 2) Luna^®^ HILIC column (50 × 2.0 mm, 3 μm), 3) YMC Hydrosphere™ C18 column (50 × 2.0 mm, 3 μm), 4) Halo™ C18 column (100 × 2.1 mm, 2.7 μm), and 5) Gemini^®^ C18 column (50 × 2.0 mm, 3 μm). Among the tested columns, the best peak shape was obtained with the Gemini^®^ C18 column at an adequate retention time. In addition, the optimal column temperature and mobile phase flow rate were determined by varying the temperatures at 35, 40, 45, and 50 °C and the flow rates at 0.1, 0.2, 0.3, and 0.4 mL/min, respectively, with respect to the chromatographic separation, peak shapes, and method reproducibility. The best separation results were obtained at the column temperature of 40 °C and the flow rate of 0.2 mL/min. In order to determine the optimal composition of the mobile phase, diverse combinations of solvents were investigated as the potential mobile phase. Use of acetonitrile and 0.1% formic acid in distilled water as the mobile phase resulted in an excessively high baseline and peak interference between the analyte and IS in the tested matrix. With the incrementally increasing proportion of water in the mobile phase, the chromatographic baseline rose. The baseline dropped substantially, and peak interference disappeared with the change of the mobile phase to methanol and 10 mM ammonium acetate. The optimal proportion of methanol in the mobile phase was determined based on the peak shape and resolution. Overall, the best chromatographic separation results were obtained with 90% methanol, compared to 80% in the mobile phase. Thus, a Gemini C18 column with the mobile phase of 10 mM ammonium acetate-methanol (10:90, *v/v*) at a flow rate of 0.2 mL/min produced the best peak shape, optimal sensitivity, good resolution, and short run time (2.5 min) of the analyte.

### 2.2. Method Validation

#### 2.2.1. Selectivity and Sensitivity

**Figure 2:**
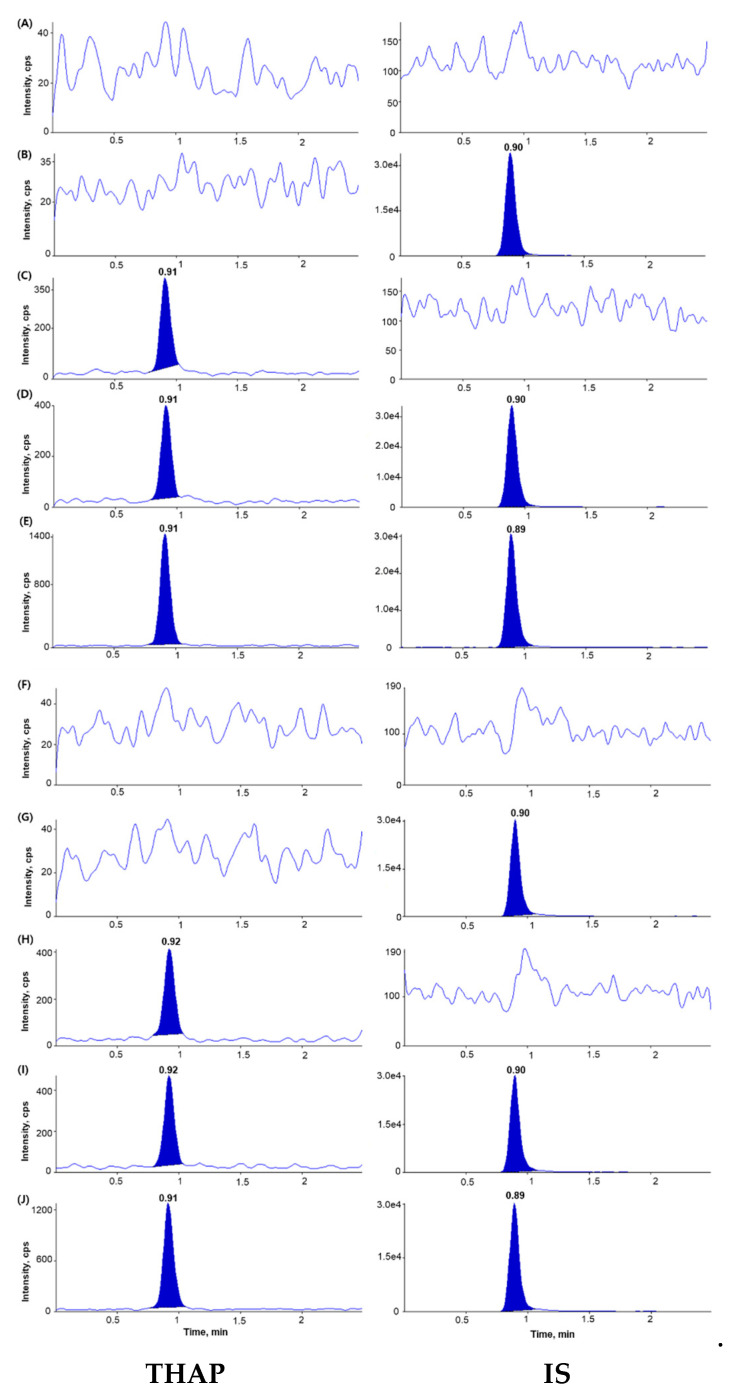
Representative chromatograms of: (**A**) blank rat plasma; (**B**) blank rat plasma spiked with IS (2 µg/mL); (**C**) blank rat plasma spiked with THAP (0.1 µg/mL, lower limit of quantitation (LLOQ)); (**D**) blank rat plasma spiked with THAP (0.1 µg/mL, LLOQ) and IS (2 µg/mL); (**E**) rat plasma sample of R06 12 h after oral administration of THAP 500 mg/kg (measured concentration 0.374 µg/mL); (**F**) blank dog plasma; (**G**) IS-spiked (2 µg/mL) dog plasma; (**H**) THAP-spiked (0.1 µg/mL, LLOQ) blank dog plasma; (**I**) blank dog plasma spiked with both THAP (0.1 µg/mL) and IS (2 µg/mL); and (**J**) dog plasma sample of R05 24 h after oral administration of THAP 500 mg immediate-release tablet (measured concentration 0.302 µg/mL). Panels on the left side were chromatograms for THAP while those on the right side were for IS.

The chromatograms of blank plasma, blank plasma spiked with IS (2 µg/mL), blank plasma spiked with THAP (0.1 µg/mL), and blank plasma spiked with both THAP (0.1 µg/mL) and IS (2 µg/mL) showed no interfering peaks at the retention times of the analyte and IS, indicating adequate selectivity of the newly developed analytical method (Figure 2). The lower limit of quantitation (LLOQ) was 0.1 µg/mL for THAP with signal-to-noise (S/N) ratios > 10, suggesting a sufficiently sensitive method to quantitate THAP in plasma after the oral administration of THAP to rats and dogs.

#### 2.2.2. Linearity and Carry-over

All calibration curves plotted with seven THAP concentrations in the range of 0.1–100 µg/mL were linear for both rat and dog plasma samples. The linear regression equation for the THAP calibration curves (*n* = 4) with the mean ± standard deviation (SD) of the slope and the intercept was as following Equation (1) in rat plasma:

*y* = 0.125 (± 0.008) *x* − 0.000451 (± 0.001703) (*r*^2^ ≥ 0.9995) (1)

*y* = 0.150 (± 0.002) *x* − 0.002042 (± 0.001337) *(r^2^ ≥* 0.9987) (2)

and as Equation (2) in dog plasma, respectively. The SDs of all concentrations were within ± 10%, demonstrating adequate reproducibility of the calibration curves (Table S1). Negligible carry-over was observed.

#### 2.2.3. Precision and Accuracy

As shown in Table 1, the within- and between-day coefficients of variation (CVs) were < 15% in both rat and dog plasma. Additionally, the intra- and inter-run accuracy was within ± 15% from the tested nominal concentrations in both rat and dog plasma. Overall, our precision and accuracy test results confirmed that the newly developed analytical assay is accurate and precise to measure THAP concentrations in rat and dog plasma.

#### 2.2.4. Extraction Recovery and Matrix Effects

Sample preparation is one of the most critical steps in the biomedical analysis to substantially eliminate interfering substances and improve the assay sensitivity (i.e., lower LLOQ). In this present study, a one-step protein precipitation (PP) procedure was employed with acetonitrile as a solvent to extract the analytes from both rat and dog plasma samples. The mean extraction recovery for THAP in rat and dog plasma was all greater than 90% at all three tested quality control (QC) concentrations with the CVs of 15% or less (Table 2), suggesting high reproducibility and excellent analyte recovery using our one-step sample preparation method with PP.

According to previous studies, the effects of the matrix on extraction recovery, ion enhancement, and ion suppression should be critically assessed to develop a valid bioanalytical method using LC-MS/MS [15,16]. As shown in Table 2, the mean matrix effects for THAP in rat and dog plasma ranged from 102.34% to 113.59% with the corresponding CV (%) values of 15% or less at the three QC concentrations. Thus, the quantitative analytical method developed in this present study was deemed to be free of substantial matrix effects between the analyte and endogenous substances in rat and dog plasma.

#### 2.2.5. Stability

The mean percentage peak areas of the stock solution at room temperature for 7 h to those of the freshly prepared stock solution were 102.15% at 0.3 µg/mL and 100.92% at 80 µg/mL. In rat and dog plasma, THAP was stable under all experimental stability conditions as shown in Table 3, including at room temperature, 4 °C, and −70 °C for up to 7 h (short-term); over five freeze-thaw cycles (99.62–102.84% in rat plasma and 98.41–105.31% in dog plasma); in the autosampler at 10 °C for 44 h (100.58–105.00% in rat plasma and 97.58–106.75% in dog plasma); and at −70 °C for 56 days in rat plasma (99.39–111.67%) and for 32 days in dog plasma (99.45–103.95%) (long-term stability). Therefore, based on the deviations from the nominal concentration within ± 15%, THAP was considered stable in rat and dog plasma under all examined conditions without substantial degradation.

#### 2.2.6. Validation of Dilution

At the THAP dose of 500 mg/kg and 750 mg/kg administered to rats, plasma THAP concentrations close to the peak concentration in the rats were greater than 100 µg/mL (upper limit of quantification [ULOQ]). Those samples with initially measured concentrations exceeding the ULOQ were diluted three-fold to determine their concentrations within the calibration curve concentration range. Therefore, dilution integrity was validated with five replicates of THAP-spiked plasma samples diluted by a factor of three with blank rat plasma to the original QC concentrations of 0.3, 25, and 80 µg/mL. The accuracy and precision of dilution integrity for rat plasma samples satisfied the validation criteria, which is defined as the deviations from the nominal concentrations within ± 15% (Table S2), suggesting reliability of the dilution method for samples with concentrations initially determined to be above the ULOQ.

### 2.3. Application to a Pharmacokinetic Study in Rats and Dogs

**Figure 3:**
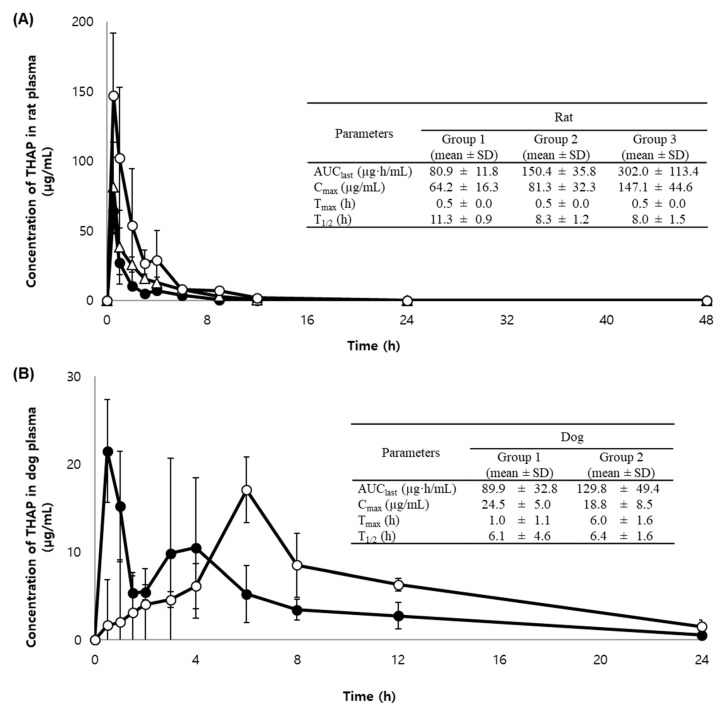
Mean (± SD) plasma concentration–time profile of THAP in the plasma samples of (**A**) rats after the oral administration of 250 mg/kg (●; Group 1), 500 mg/kg (△; Group 2), or 750 mg/kg (○; Group 3) THAP as aqueous solution and (**B**) dogs after the oral administration of 500 mg THAP as immediate-release (●; Group 1) or sustained-release tablet (○; Group 2). Pharmacokinetic parameters are summarized in the inset table. The data point and error bars represent mean and SD (*n* = 5).

Our newly developed and validated bioanalytical method was successfully employed in a pharmacokinetic study of orally administered THAP to analyze a total of 275 plasma samples from 15 rats receiving THAP in deionized water containing 2% carboxymethyl cellulose (CMC) and from 10 dogs receiving immediate-release (IR) or sustained-release (SR) THAP tablets. The rat plasma samples with concentrations initially measured to be higher than the ULOQ were diluted and re-analyzed as described in Section 3.7. Figure 3 shows the mean ± SD plasma concentration-time profiles and pharmacokinetic parameters of THAP orally administered to rats and dogs. Noncompartmental methods were used to calculate the following pharmacokinetic parameters of THAP (Figure 3): the area under the plasma concentration–time curve from time zero to the time of the last measurable concentration (AUC_last_), the peak plasma concentration (C_max_), the time to reach the C_max_ (T_max_), and the terminal half-life (T_1/2_). As shown in Figure 3A, the AUC_last_ and C_max_ of THAP increased in a dose-dependent manner in rats that were orally given the aqueous solution formulation of THAP.

Comparing the pharmacokinetic profiles of IR and SR tablets of THAP orally administered to dogs (Figure 3B), delayed T_max_ and slightly lower C_max_ were observed with the SR tablet compared to the IR tablet as expected. The prolonged T_max_ from 1 h (IR tablet) to 6 h (SR tablet) is consistent with our dissolution test results. Up to 80% of THAP was released from IR tablets in 0.25 h and from SR tablets in 6 h (data not shown). Notably, the SR tablet produced significantly higher AUC_last_ by approximately 40% compared to the IR tablet. In addition, a bi-modal peak was observed with the IR tablet, but not with the SR tablet, based on the plasma concentration-time profile of THAP in dogs. The time course of plasma concentrations of THAP in dogs may suggest that THAP is absorbed twice in the stomach as well as in the upper small intestine region. Considering the time needed for a tablet to be disintegrated, the IR tablet dosage form absorbed twice in two different areas of the gastrointestinal tract may result in the double-peak phenomenon [17,18]. Similarly, a slightly doubled peak was also observed at the highest dose group of THAP in rats (750 mg/kg). At this high dose, the drug is mostly absorbed in the stomach, and the remaining fraction of the drug is transferred to and absorbed in the intestinal area.

Overall, our newly developed and validated biomedical assay using LC-MS/MS was successfully applied to accurately and precisely quantitate THAP in biological samples after the oral administration of THAP liquid formulation to rats and IR or SR tablets to dogs.

### 2.4. Incurred Sample Reanalysis (ISR)

ISR was conducted to assess the reproducibility of the quantitative analytical method newly developed in our present study. According to the regulatory guidelines [19,20], the bioanalytical method was deemed to be reproducible if the deviations between the original measurements and the reanalysis results were within ± 20% for at least two thirds of the reanalysis samples. Among a total of 34 samples re-analyzed, 94.12% and 100.0% of rat and dog samples, respectively, satisfied the pre-specified regulatory ISR acceptance criteria (Table 4).

## 3. Materials and Methods

### 3.1. Chemicals and Reagents

THAP (purity 100%) and 2′,4′,6′-trihydroxybenzaldehyde (IS, purity 99.9%) were obtained from Sigma-Aldrich (St. Louis, MO, USA). Acetonitrile and methanol of high-performance liquid chromatography (HPLC)-grade were purchased from J.T. Baker (Phillipsburg, NJ, USA). An Aqua MAX™-Ultra (Young Lin Instrument, Gyeonggi-do, Korea) water purification system was used to distill water for HPLC analysis. All other chemicals and solvents used were of the highest analytical grades available.

### 3.2. Animals

Healthy Sprague Dawley rats (*n* = 15, mean ± SD weight 261.4 ± 6.8 g) and beagle dogs (*n* = 10, mean ± SD weight 9.85 ± 0.60 kg) were purchased from Orient Bio Inc. (Seongnam, Korea). The animals were kept in a light-controlled room at 22 ± 2 °C with relative humidity of 55 ± 5% in the Animal Center of Korea Preclinical Center (Gyeonggi-do, Korea). The pharmacokinetic study protocols (KPC-E2019099 for rats and KPC-M2019046 for dogs) were approved by the Animal Care and Use Committee of the Korea Preclinical Center (Gyeonggi-do, Korea).

### 3.3. Instruments and Analytical Conditions

Chromatographic separation was performed using an Agilent 1200 Series HPLC (Agilent Technologies, Inc., Palo Alto, CA, USA) system with a Gemini C18 column (50 × 2.0 mm, 3 µm, Phenomenex, Torrance, CA, USA) protected by a guard column at 40 °C. The mobile phase composed of 10 mM ammonium formate in water and methanol (10:90, *v/v*) was isocratically pumped into the HPLC system at a flow rate of 0.2 mL/min with a total run time of 2.5 min. The column was eluted into an API 4000 triple quadrupole mass spectrometer (Applied Biosystems, Framingham, Massachusetts, USA), operating in the multiple reaction monitoring (MRM) mode with a negative ion electrospray source. The optimized instrument settings were as follows: curtain gas flow of 20 L/h; collision gas flow of 6 L/h; ion source gas 1 pressure of 50 psi; ion source gas 2 pressure of 60 psi; ion spray voltage of −4500 V; and temperature of 500 °C. The precursor ions of THAP and IS were generated using declustering potentials of −100 and −75 V, respectively, and they were fragmented at a collision energy of −28 and −24 eV, respectively. The molecular mass transitions from precursor to product ion were optimized at *m/z* 166.89 > 82.8 for THAP and at 152.89 > 82.8 for IS. Data were processed using Analyst^®^ program (Ver. 1.6.2, AB SCIEX, Toronto, ON, Canada).

### 3.4. Preparation of Calibration Standards and QC Samples

Stock solutions of THAP and IS were prepared in 100% acetonitrile at the concentration of 5 mg/mL and 2 mg/mL, respectively. The stock solution of THAP was further diluted with 50% acetonitrile to obtain working standard solutions at the concentrations of 1, 5, 10, 50, 250, 500, and 1000 µg/mL. Calibration standard samples were prepared from THAP working standard solutions spiked with blank rat or dog plasma to final concentrations of 0.1, 0.5, 1, 5, 25, 50, and 100 µg/mL. QC samples of THAP were independently prepared in the similar manner at concentrations of 0.3, 25, and 80 µg/mL. The IS solution at the concentration of 2 µg/mL was also prepared using 50% acetonitrile. All of the prepared solutions were stored at −20 °C. The calibration standards and QC samples were freshly prepared on each day of analysis.

### 3.5. Sample Preparation

Plasma samples were stored in a deep freezer at −70 °C and thawed at room temperature. An aliquot of each plasma sample (10 μL) was transferred to 1.75 mL microtubes (Axygen Scientific, Inc., California, USA). In each microtube, 20 μL of IS (2 μg/mL) and 500 μL of 100% cold acetonitrile were added and then vortexed for 10 min. After centrifugation at 20,800× *g* for 15 min, 50 μL of the supernatant was pipetted into a 2.0 mL microtube and diluted with 350 μL of 50% acetonitrile (*v/v*). After being vortexed for 10 min and centrifuged at 20,800× *g* at 4 °C for 10 min, a 2 μL aliquot was injected into the LC-MS/MS system for quantitation.

### 3.6. Method Validation in Rat and Dog Plasma

The bioanalytical method was validated for selectivity, LLOQ, linearity, precision, accuracy, recovery, matrix effect, carry-over, stability, and dilution effects in compliance with the FDA [19] and the MFDS Bioanalytical Method Validation Guidelines [20]. Throughout the validation study, heparin was used as an anticoagulant for plasma samples.

#### 3.6.1. Selectivity and LLOQ

Selectivity was evaluated by analyzing six randomly selected blank plasma samples from different sources to examine potentially interfering endogenous substances concurrently eluted at the retention times of THAP and IS. The LLOQ was determined as the lowest quantifiable concentration with a signal to noise (S/N) ratio of 10 or greater.

#### 3.6.2. Linearity and carry-over

Linearity of the calibration curves (*y* = *ax* + *b*) was validated utilizing seven concentrations of THAP over the range of 0.1 µg/mL to 100 µg/mL by plotting the ratios of the peak area of THAP to that of IS (*y*) versus THAP concentrations (*x*) using a linear regression model weighted with 1/*x*^2^. Carry-over was assessed by analyzing a blank sample following the ULOQ calibration standard sample.

#### 3.6.3. Precision and Accuracy

Between- and within-run precision and accuracy were examined using five replicates at four concentrations (0.1, 0.3, 25, and 100 µg/mL) on three consecutive days. Precision and accuracy were evaluated as %CV and relative error (RE), respectively. Accuracy and precision were considered acceptable if deviations from the theoretical concentration were within ± 15% except for LLOQ, where they should be within ± 20%.

#### 3.6.4. Extraction Recovery and Matrix Effects

Extraction recovery and matrix effects were assessed for both rat and dog plasma by comparing the peak area ratios of QC samples spiked with THAP before extraction [A], after extraction [B], and the pure standard solution in 50% acetonitrile (*v/v*) [C] at three QC concentrations (0.3, 25, and 80 µg/mL, *n* = 6). The recovery of THAP and IS was measured at three QC concentrations by estimating the peak area ratios of [A] to [B]. The matrix effect was determined by calculating the peak area ratios of [B] to [C].

#### 3.6.5. Stability

The stability of THAP in rat and dog plasma was examined under the following conditions at concentrations of 0.3, 25, and 80 µg/mL (*n* = 3): freeze-thaw stability after five freeze-thaw cycles at −70 °C; short-term stability at room temperature, 4 °C, and −70 °C for 7 h; autosampler stability at 10 °C for 44 h; and long-term stability at −70 °C for 56 days and 32 days in rat and dog plasma, respectively. The stability of the stock solution at low (0.3 µg/mL) and high (80 µg/mL) QC concentrations (*n* = 3) stored at room temperature for 7 h was also tested by comparing their peak areas to those of freshly prepared stock solutions.

#### 3.6.6. Dilution Integrity in Rat Plasma

Rat plasma samples with the THAP concentration above the ULOQ (100 μg/mL) were diluted with the same matrix (blank rat plasma) and were re-analyzed. To validate the dilution method, samples spiked with the THAP concentrations 3-fold higher than the QC concentrations (i.e., 0.3, 25, and 80 µg/mL) were prepared as described in Section 3.4. The samples were then diluted three times with the blank rat plasma to the original QC concentrations and were pretreated as depicted in Section 3.5. The precision and accuracy were estimated in terms of CV (acceptable range: under 15%) and the percentage deviation from the theoretical concentrations (acceptable range: within *±* 15%), respectively.

### 3.7. Application to a Pharmacokinetic Study

Male rats orally received a single dose of THAP (250, 500, or 750 mg/kg) dissolved in deionized water containing 2% CMC by gavages. Serial blood samples (0.22 mL each) were collected via the right jugular vein immediately before (0 h) and at 0.5, 1, 2, 3, 4, 6, 9, 12, 24, and 48 h (*n* = 5 per each sample time) post-dosing. Male dogs received a single oral dose of THAP tablet (500 mg) as either an immediate-release tablet (Group 1) or a sustained-release tablet (Group 2) (Table 5). Blood samples (1.0 mL) were drawn from a median vein of a forelimb at the following time points after the administration of THAP: 0.5, 1, 1.5, 2, 3, 4, 6, 8, 12, and 24 h (*n* = 5 per each sample time). After sampling blood from the animals, the cannula was flushed with an equal volume of heparinized saline solution (50 U/mL) to prevent coagulation and to replace the blood loss. Heparin was used as an anticoagulant throughout the study procedure. Plasma was harvested by centrifugation at 4 °C, 1700× *g* for 10 min and then stored at −70 °C before quantification. Noncompartmental analysis was performed to calculate the AUC_last_ and T_1/2_ of THAP using WinNonlin version 8.1 (Pharsight Corporation, Mountain View, CA). The C_max_ and T_max_ of THAP were determined based on the individual plasma THAP concentration–time profiles.

### 3.8. ISR

Incurred sample reanalysis was conducted to evaluate the reproducibility of our newly developed bioanalytical method using 34 selected samples (17 samples each from the rats and dogs) near the C_max_ and at the elimination phase of the pharmacokinetic profile. Measured THAP concentrations were compared between the initial and reanalyzed data. The deviations of the reanalyzed data from the original measurements should not be more than ± 20% [21].

## 4. Conclusions

This study describes the development and validation of a simple, rapid, and reproducible analytical method to determine THAP concentrations in rat and dog plasma. The newly devised method satisfies all of the validation criteria suggested in the bioanalytical method validation guidelines from the FDA as well as the MFDS [19,20], demonstrating specificity, reliability, and reproducibility of the established quantitation method over the concentration range. Our rapid bioanalytical method with a simple pretreatment procedure may be suitable for the routine, high-throughput quantitative analysis with a large number of samples.

## Figures and Tables

**Table 1:** Intra- and inter-day precision and accuracy for the LC-MS/MS method to determine THAP in rat and dog plasma (*n* = 5).

	Theoretical Concentration (µg/mL)	Predicted Concentration (µg/mL) (Mean ± SD)						Precision (CV %) ^a^		Accuracy (%) ^b^	
		Intra-Day			Inter-Day			Intra-Day	Inter-Day	Intra-Day	Inter-Day
Rat	0.1	0.10	±	0.01	0.09	±	0.01	10.18	9.86	103.80	98.80
	0.3	0.28	±	0.01	0.30	±	0.02	1.84	5.50	94.33	99.40
	25	25.06	±	0.22	25.76	±	0.62	0.86	2.39	100.24	103.02
	100	99.02	±	1.83	98.49	±	1.32	1.85	1.34	99.02	98.49
Dog	0.1	0.10	±	0.01	0.11	±	0.01	3.86	9.73	97.00	105.00
	0.3	0.31	±	0.01	0.31	±	0.01	4.00	4.18	102.60	103.76
	25	24.88	±	0.19	25.74	±	0.92	0.77	3.56	99.52	102.97
	100	94.46	±	0.81	94.47	±	1.07	0.86	1.14	94.46	94.07

^a^ coefficients of variation (CV) (%) = (standard deviation of calculated concentrations/mean concentration) × 100; ^b^ Accuracy (%) = (predicted concentration/nominal concentration) × 100.

**Table 2:** Extraction recovery and matrix effects of THAP in rat and dog plasma.

	Nominal Concentration (µg/mL)	Extraction Recovery ^a^ (%)				Matrix Effect ^b^ (%)			
		Mean ± SD			CV	Mean ± SD			CV
Rat									
THAP	0.3	90.00	±	5.08	5.64	109.29	±	5.46	5.00
	25	97.16	±	1.69	1.74	102.34	±	2.45	2.40
	80	97.47	±	1.90	1.95	102.34	±	1.82	1.78
IS	2	82.39	±	2.51	3.05	86.81	±	2.86	3.29
Dog									
THAP	0.3	94.40	±	2.54	2.69	113.59	±	2.78	2.44
	25	103.81	±	2.43	2.34	103.64	±	3.63	3.50
	80	102.51	±	2.38	2.32	103.76	±	2.57	2.48
IS	2	70.75	±	3.83	5.42	90.86	±	3.62	3.98

^a^ Extraction recovery (%) = [(peak area of analyte spiked before extraction)/(peak area of analyte spiked after extraction)] × 100. ^b^ Matrix effect (%) = [(peak area of analyte spiked after extraction)/(peak area of analyte in the pure standard solution)] × 100. Data are presented as mean ± standard deviation (SD) (*n* = 6).

**Table 3:** Stability data for THAP in rat and dog plasma samples (*n* = 3).

Nominal Concentration (µg/mL)		Plasma Stability (Mean %)					
	Species	Room Temperature (7 h)	4 °C (7 h)	−70 °C (7 h)	Freeze-Thaw Stability (5 Cycles)	Autosampler (44 h, 10 °C)	Long-Term ^a^ (−70 °C)
	Rat						
0.3		98.22	99.22	98.11	101.00	105.00	111.67
25		101.62	106.91	109.87	102.84	104.80	104.15
80		100.47	100.98	101.61	99.62	100.58	99.39
	Dog						
0.3		109.89	111.89	109.56	101.33	99.67	102.89
25		106.66	105.57	105.88	105.31	106.75	103.95
80		98.17	98.87	97.32	98.41	97.58	99.45

^a^ 56 and 32 days in rat and dog plasma, respectively.

**Table 4:** Incurred sample reanalysis (ISR) result summary of THAP in rats and dogs.

	Selected Sample Numbers	Accepted Sample Numbers	Results (%)
Rat	17	16	94.12
Dog	17	17	100.00

**Table 5:** The compositions of IR and SR THAP tablets for dogs.

Composition	Function *	IR Tablet (mg)	SR Tablet (mg)
THAP	API	500	500
Hydroxypropyl cellulose (HPC)	Binder	20	20
Kollidon SR (Soluplus^®^)	SR material	-	250
Microcrystalline cellulose	Diluent	200	-
Sodium starch glycolate	Disintegrant	23	23
Colloidal silicon dioxide	Glidant	15	15
Sodium stearyl fumarate	Lubricant	8	8
Total Weight		766	816

* API indicates active pharmaceutical ingredient. HPC was supplied from Ashland Inc. (Kentucky, US) and kollidon SR from BASF (Ludwigshafen, Germany). Microcrystalline cellulose, sodium starch glycolate, colloidal silicon dioxide, and sodium stearyl fumarate were purchased from Hwail Pharm (Seoul, Republic of Korea).

## Supplementary Materials

The followings are available online. Table S1: Linearity from the regression analysis of our bioanalytical method to quantify THAP in rat and dog plasma., Table S2: Results of 3-fold dilution integrity experiment of THAP in rat plasma (*n* = 5).
