# Supplementary material for: Development and Validation of an LC-MS/MS Assay to Quantitate 2′,4′,6′-Trihydroxyacetophenone in Rat and Dog Plasma and its Application to a Pharmacokinetic Study
Source: Molecules. 2020 Sep 23;25(19):4373. doi: 10.3390/molecules25194373 (PMC7583961; doi:10.3390/molecules25194373)
Supplement: Supplementary file 1 [file molecules-25-04373-s001.pdf]

Table S1. Linearity obtained after regression analysis of the method for determining THAP in rat and dog plasma.

	Number	Slope	Intercept	*r*
Rat				
	1	0.122	-0.0003480	0.9995
	2	0.116	0.0013700	0.9997
	3	0.129	-0.0000847	0.9995
	4	0.133	-0.0027400	0.9997
Dog				
	1	0.153	-0.0018800	0.9990
	2	0.149	-0.0039700	0.9993
	3	0.150	-0.0009880	0.9993
	4	0.149	-0.0013300	0.9987

Table S2. Results of 3-fold dilute validation experiment of THAP in rat plasma (n = 5).

Nominal Concentration (μg/mL)	Dilution factor 3		
	Mean ± SD (μg/mL)	CV (%)	Accuracy (%)
0.3	0.30 ± 0.01	2.58	100.40
25	26.42 ± 0.22	0.84	105.66
80	278.42 ± 1.54	1.97	98.03
